# Lifespan Extension by the Antioxidant Curcumin in Drosophila Melanogaster

**Published:** 2006-12

**Authors:** Brianne K. Suckow, Mark A. Suckow

**Affiliations:** *400 Freimann Life Science Center, University of Notre Dame, Notre Dame, IN 46556, USA*

**Keywords:** curcumin, atoxdant, lifespan, drosophila, disulfiram

## Abstract

The interest in health benefits associated with consumption of anti-oxidants has led to investigations examining the possibility that diets rich in anti-oxidants promote lifespan extension. Studies using the standard fruit fly (Drosophila melanogaster) model of longevity have shown that the antioxidants vitamin E and N-acetyl cysteine prolong lifespan. Turmeric is a spice which has been consumed and used for medicinal purposes for many centuries in Asia. Interestingly, turmeric contains the powerful antioxidant, curcumin. To test the hypothesis that dietary curcumin prolongs lifespan, groups of 30 male *D. melanogaster* were cultured on media containing 1) no additive; 2) 0.5 mg of curcumin/gram of media; 3) 1.0 mg of curumin/gram of media; 4) 1.0μg of the superoxide dismutase inhibitor, disulfiram/gram of media; 5) 10 g of disulfiram/gram of media; 6) 0.5 mg curcumin and 1.0 g disulfiram/ gram of media; 7) 1.0 mg curcumin and 1.0 g disulfiram/ gram of media; 8) 0.5 mg curcumin and 10 g disulfiram/gram of media; or 9) 1.0 mg curcumin and 10 g disulfiram/gram of media. The number of live fruitflies was noted daily and mean lifespan determined for each treatment group. A significant (*P*≤0.05) increase in mean lifespan was noted only for the fruitflies maintained on 1.0 mg of curcumin/gram of media; this effect was reversed by addition of disulfiram. These results demonstrate that dietary curcumin prolongs lifespan and that this effect is associated with enhanced superoxide dismutase activity.

## INTRODUCTION

Dietary anti-oxidants have been associated with a variety of health benefits, including prevention of heart disease and cancer. For example, oral administration of curcumin to rodents has been shown to prevent cancer in the colon, skin, stomach, liver, lung and breast (1). In one study, low doses of curcumin were administered to patients with advanced colorectal cancer for up to 4 months. Of these patients, 30% experienced disease stabilization for three months or longer compared to no stabilization in patients not treated with curcumin (2).

The scavenging of oxygen radicals is an important mechanism in the prevention of cardiovascular disease, and it is believed that these benefits derive from antioxidants which inactivate oxygen radicals such as superoxide anion generated during the course of normal metabolism via a reaction which converts superoxide to hydrogen peroxide and oxygen (3-7). Such oxygen radicals are capable of causing DNA damage and oxidation of cell membrane and cytoplasmic components, thereby crippling cells in a way not conducive to survival of the organism.

The interest in health benefits associated with consumption of anti-oxidants has led to investigations examining the possibility that diets rich in anti-oxidants promote lifespan extension. Studies using the standard fruit fly (*Drosophila melanogaster*) model of longevity (8) have shown that the antioxidants vitamin E and N-acetyl cysteine prolong lifespan (9, 10).

Turmeric is a spice derived from the turmeric plant (*Curcuma longa*) and has been consumed and used for medicinal purposes for many centuries in Asia (11). For example, turmeric has been used topically as a treatment for wounds, inflammation, and tumors. Interestingly, turmeric contains the powerful antioxidant, curcumin. Curcumin has been extensively studied for over 30 years and its antioxidant and anticancer properties are well known (12-14). Specifically, curcumin has been shown to scavenge superoxide anion and nitric oxide (15, 16). Dietary supplementation of curcumin to gerbils was able to protect against brain damage following experimentally induced cerebral ischemia, a process mediated by lipid peroxidation (17). In spite of substantial evidence demonstrating the powerful antioxidant ability of curcumin, no studies examining the ability of that compound to prolong life have been conducted. The studies described here were undertaken to evaluate the effect of dietary cucumin on lifespan in *D. melanogaster* and to establish the mechanism of this effect.

## MATERIAL AND METHODS

### Test Organisms

Wild 1-A wild type fruit flies (*Drosophila melanogaster*) were acquired from the Bloomington Drosophila Stock Center at Indiana University and maintained on commercial fruit fly culture media. Upon receipt, fruit flies were kept together for three days, long enough to mate and produce eggs within the media. After three days, the adult flies were removed. All cultures were maintained at room temperature under normal room lighting conditions. Within ten days, the eggs had hatched; larvae pupated and developed into adult flies.

### Chemical Compounds

Curcumin and disulfiram (an inhibitor of superoxide dismutase) were obtained from Sigma Chemical Co., St. Louis, MO.

### Experimental Design

Male fruit flies were transferred to containers containing either media only (Group 1) or the following additives; Group 2) 0.5 mg of curcumin/gram of media; Group 3) 1.0 mg of curumin/gram of media; Group 4) 1.0 μg of disulfiram/gram of media; Group 5) 10 μg of disulfiram/gram of media; Group 6) 0.5 mg curcumin and 1.0 μg disulfiram/gram of media; Group 7) 1.0 mg curcumin and 1.0 μg disulfiram/gram of media; Group 8) 0.5 mg curcumin and 10 μg disulfiram/gram of media; or Group 9) 1.0 mg curcumin and 10 μg disulfiram/gram of media. For each treatment, there were three cultures, each with ten fruitflies (total of 30 fruit flies/treatment). Each day, the number of live fruit flies was counted, and from this the mean life span for each type of culture was calculated. One-way analysis of variance with Tukey’s post-hoc test was used to determine if the differences between treatments was significant, with differences considered to be significant at *P*≤0.05.

## RESULTS

### Effect of curcumin on lifespan

**Figure 1:**
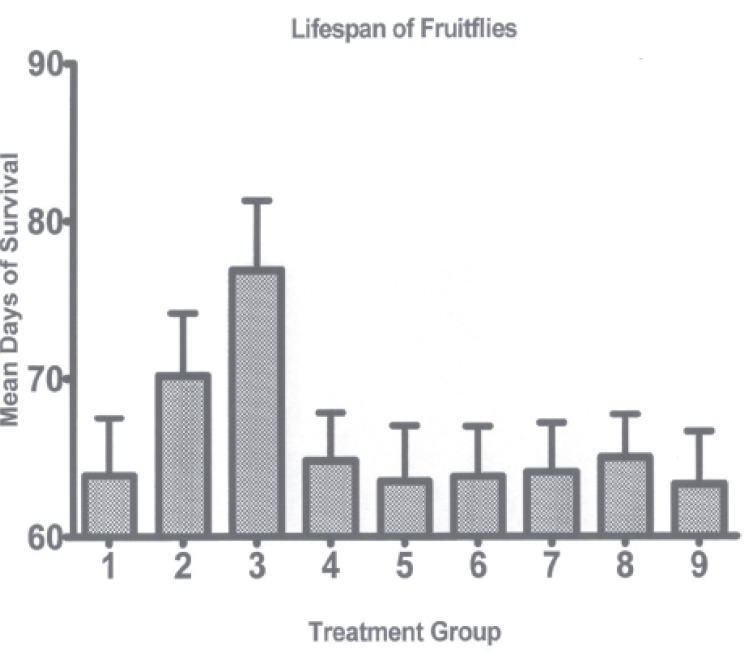
Lifespan of fruitflies. Treatment groups are media with no additive (Group 1); 0.5 mg of curcumin/gramof media (Group 2); 1.0 mg of curcumin/gram of media (Group 3); 1.0 mg of disulfiram/gram of media (Group 4); 10 mg of disulfiram/gram of media (Group 5); 0.5 mg of curcumin and 1.0 mg of disulfiram/gram of media (Group 6); 1.0 mg of curcumin and 1.0 mg of disulfiram/gram of media (Group 7); 0.5 mg of curcumin and 10 mg of disulfiram/gram of media (Group 8); and 1.0 mg of curcumin and 10 mg of disulfiram/gram of media (Group 9). There were no significant differences (*P*<0.05) in mean lifespan between treatment groups except or an increased lifespan in fruitflies maintained on media containing 1.0 mg of curcumin per gram of media. Note that the baseline is set at 60 days.

Figure 1 shows the effect of dietary curcumin on lifespan in *D. melanogaster*. The mean lifespan of control fruitflies maintained on media with no additive (Group 1) was 63.9 days. In contrast, a significant (*P*≤0.05) increase in mean lifespan to 76.9 days was noted in fruit flies maintained on media supplemented with 1.0 mg curcumin/gram of media (Group 3). No other treatments resulted in significantly increased or decreased mean life span compared to control fruit flies.

### Potential role of superoxide dismutase on lifespan extension by curcumin

Since disulfiram inhibits superoxide dismutase, it could be expected that if the increase in lifespan resulting from curcumin consumption is mediated by an increase in superoxide dismutase activity, that disulfiram treatment could reverse this effect. Indeed, a mean lifespan of 64.1 days and 63.3 days was found in fruit flies maintained on media with the high dose of curcumin with either 1.0 μg or 10 μg of disulfiram (Groups 7 and 9), respectively; neither of these values was significantly different from the control fruit flies (Group 1) but both were significantly less than the lifespan of fruit flies maintained on media containing 1.0 mg curcumin/gm of media (Group 3).

## DISCUSSIONS

*D. melanogaster* has been extensively used to study death as the eventual outcome of processes that harm the organism and which collectively constitute aging (8). Reactive oxygen species, such as superoxide anion, are the byproducts of oxidative metabolism and are believed to be the principle cause of aging. Indeed, antioxidant compounds such as vitamin E and N-acetylcysteine which scavenge oxygen radicals are associated with increased lifespan in fruit flies (9, 10).

A principle way for organisms to avoid damage by oxygen radicals is via the action of the enzyme, superoxide dismutase which facilitates the conversion of superoxide anion to less damaging compounds. That this is an important mechanism in longevity of the fruit fly is highlighted by a number of studies. The genome of *D. melanogaster* has been shown to have one region that can enhance superoxide dismutase activity and four regions that can suppress it (18). This finding suggests that within a given population of fruit flies there exists genetic diversity to accommodate changing environmental conditions that might result in either greater or lesser oxygen radical generation. Further, genetic disruption of superoxide dismutase in *D. melanogaster* is associated with reduced lifespan (19, 20). Over-expression of superoxide dismutase resulted in increased life span of *D. melanogaster* (21, 22).

Curcumin is a safe compound, routinely consumed as a component of the spice, turmeric. Curcumin has been shown to be a powerful inhibitor of oxygen radicals and inflammation (23, 24). The study presented here showed that dietary curcumin extended the lifespan of *D. melanogaster* in a dose-dependent fashion, suggesting that greater inhibition of oxygen radicals resulting from a higher concentration of curcumin was associated with lifespan extension. In other words, while 1.0 mg of curcumin/gram of media was sufficient to extend the lifespan, 0.5 mg of curcumin/gram of media was not. This data is consistent with the idea that consumption of anti-oxidants is capable of inhibiting processes which limit lifespan.

The mechanism of action for *D. melanogster* lifespan extension by curcumin was uncertain, though a role for superoxide dismutase was suspected. Disulfiram is a compound shown to inhibit superoxide dismutase (25, 26). Our data show that when disulfiram was added, the ability of 1.0 mg of curcumin/gram of media to extend lifespan was reversed. Though there may also be other mechanisms involved in lifespan extension by curcumin, this result clearly shows that enhanced superoxide dismutase activity is associated with lifespan extension and may be, at least partially, involved in the mechanism of action. Direct measurement of superoxide dismutase activity is needed to confirm this role.

While no specific studies in humans have examined the association between a diet rich in turmeric (curcumin) and lifespan, the data presented here support the idea that curcumin consumption is a healthy behavior.

In summary, this research demonstrates that dietary consumption of curcumin increases lifespan in *D. melanogaster* and that the mechanism of action for this effect may be enhanced superoxide dismutase activity.
